# Coronary Air Embolism Secondary to Percutaneous Lung Biopsy: A Systematic Review

**DOI:** 10.7759/cureus.55234

**Published:** 2024-02-29

**Authors:** Shai Ring, Tusharkumar Pansuriya, Hytham Rashid, Aswin Srinivasan, Ramesh Kesavan, Skantha K Manjunath, Gnananandh Jayaraman, Siva T Sarva

**Affiliations:** 1 Department of Internal Medicine, HCA Houston Healthcare Kingwood, Houston, USA; 2 Department of Internal Medicine, Tilman J. Fertitta Family College of Medicine, University of Houston, Houston, USA; 3 Department of Pulmonary and Critical Care Medicine, HCA Houston Healthcare Kingwood, Houston, USA; 4 Department of Pulmonary and Critical Care Medicine, Tilman J. Fertitta Family College of Medicine, University of Houston, Houston, USA

**Keywords:** acute coronary syndrome, hyperbaric oxygen thetapy, in hospital cardiac arrest, coronary air embolism, percutaneous lung biopsy, systemic air emboli, right coronary artery, left lower lobe, biopsy needle, biopsy method

## Abstract

To determine mortality and morbidity associated with coronary air embolism (CAE) secondary to complications of percutaneous lung biopsy (PLB) and illicit-specific risk factor associated with this complication and overall mortality, we searched PubMed to identify reported cases of CAE secondary to PLB. After assessing inclusion eligibility, a total of 31 cases from 26 publications were included in our study. Data were analyzed using Fisher’s exact test. In 31 reported cases, cardiac arrest was more common after left lower lobe (LLL) biopsies (n=4, 80%, p=0.001). Of these patients who suffered from cardiac arrest, CAE was found more frequently in the right coronary artery (RCA) than other locations but did not reach statistical significance (n=5, 62%, p=0.39). At the same time, intervention in the LLL was significantly associated with patient mortality (n=3, 60%, p=0.010). Of the patients who died, CAE was more likely to have occurred in the RCA, but this association was not statistically significant (n=4, 57%, p=0.33). LLL biopsies have a statistically significant correlation with cardiac arrest and patient death. More research is needed to examine the effect of the air location in the RCA on patient morbidity and mortality.

## Introduction and background

Computed tomography (CT)-guided percutaneous needle biopsy is becoming a more common method to establish a histological diagnosis of lung tumors. Pneumothorax and pulmonary hemorrhage are the most common complications associated with this procedure; however, systemic air embolism (SAE) is another potentially fatal complication of CT-guided percutaneous needle biopsy, with a reported incidence of 0.02-0.07% [1, 2]. Once in left-sided circulation, air is uninhibited to embolize throughout the branches of the aorta and into general or cerebral circulation. Given the direct access to central circulation, coronary air embolism (CAE) is an even rarer subtype of SAE [3].

Albeit rare, SAE is a serious complication of CT-guided percutaneous lung biopsy (PLB) [4]. Both SAE and CAE can have catastrophic consequences with ischemic insult to end organs after entering circulation. The most precarious situations would be embolization of air into the cerebral or coronary circulations, causing stroke, myocardial infarction, and/or cardiac arrest, respectively [1]. We believe prompt clinical and radiological identification of systemic and CAE can prevent mortality and greatly lower morbidity.

Although several cases are available in the literature regarding the treatment of CAE, there are no definitive data available regarding its management. This is most likely due to the extreme rarity and under-reporting of the entity, as the typical symptoms of chest pain, shortness of breath, bradycardia, and hypotension can be non-specific [5]. This systematic review analysis aims to identify cases in the literature and evaluate patient-specific risk factors, treatments used, and how those risk factors and treatments affect the overall morbidity and mortality of CAE.

## Review

Methods

Search Strategy

**Figure 1:**
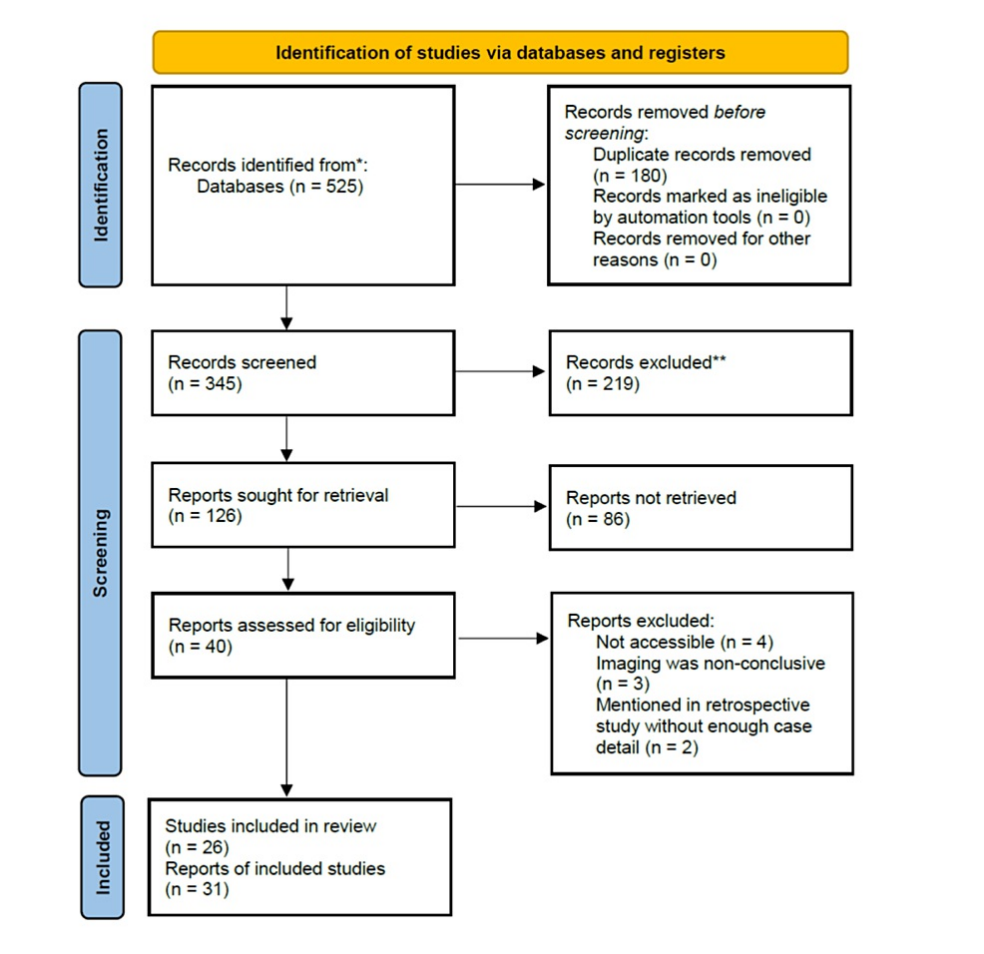
PRISMA flow diagram depicting the methods for case selection. PRISMA: Preferred Reporting Items for Systematic Reviews and Meta-Analyses. *Only one database is being used. **Lack of automated tool use.

We performed a systematic literature search for case reports from January 2001 until December 2021 pertaining to CAE secondary to PLB in PubMed. The terms used in the search criteria were as follows: air embolism and lung biopsy OR air embolism and PLB OR air embolism and transthoracic biopsy OR CAE and biopsy. Studies were assessed in accordance with the Preferred Reporting Items for Systematic Reviews and Meta-Analyses (PRISMA) guidelines for systematic review analysis (Figure 1). Initially, 525 total articles were obtained, of which 180 duplicate articles were removed. Initial screening excluded articles that did not mention PLB intervention. The second round of screening removed articles that did not mention CAE. The third round of screening removed articles with limited access, retrospective analyses without case descriptions, and cases without confirmation of CAE on imaging. The search yielded a total of 31 unique cases, which were ultimately reviewed in detail, and data were extracted regarding patient demographics, clinical manifestations, procedural details, location of embolism, treatment types, and clinical outcomes [2,6-30].

Inclusion/Exclusion Criteria

To be considered in this systematic review, patients had to have been subjected to percutaneous or transthoracic lung biopsy. Following the procedure, all patients must have developed coronary artery air emboli with or without additional extracardiac lesions. Patients who underwent PLB and had extracardiac air emboli without involvement of the coronary arteries were excluded. Subjects who failed to have CAE confirmed via CT, magnetic resonance imaging (MRI), or left heart angiography were also excluded from analysis. No exceptions were made for inclusion. Additional patients were excluded if there was restricted access to their case report or if their case was mentioned in retrospective studies without enough detail to fully analyze the case. After meeting all criteria, 31 cases were included in the study (Table 1).

**Table 1:** Baseline characteristics of cases reviewed. L: Left; R: right; M: male; F: female; LLL: left lower lobe.

Article	Age	Sex	Biopsy lesion: Left lung/right lung	Lobar location	Biopsy method	Patient position	Needle gauge
Arnold and Zwiebel [8]	60	M	L	LLL	Needle aspiration	Decubitus	19-22
Ashizawa et al. [9]	65	F	R	Not specified	Core biopsy	Supine	18 or less
Bou-Assaly et al. [10]	76	M	L	Other than LLL	Core biopsy	Decubitus	18 or less
Chang et al. [11]	73	M	R	Other than LLL	Core biopsy	Not specified	19-22
Cheng et al. [2]	35	M	L	Other than LLL	Core biopsy	Supine	18 or less
Deshmukh et al. [7]	52	M	R	Not specified	Core biopsy	Supine	19-22
El Homsi et al. [12]	57	M	L	LLL	Core biopsy	Decubitus	19-22
Franke et al. [13]	69	M	R	Not specified	Core biopsy	Prone	18 or less
Grandjean et al. [14]	62	M	R	Other than LLL	Not specified	Prone	not specified
Hare et al. [6]	67	F	L	Other than LLL	Needle aspiration	Supine	19-22
	66	M	L	Other than LLL	Core biopsy	Prone	19-22
	63	M	R	Other than LLL	Needle aspiration	Prone	19-22
Hsi et al. [15]	67	M	R	Not specified	Core biopsy	Not specified	18 or less
Hung et al. [16]	63	M	R	Not specified	Core biopsy	Prone	18 or less
Ibukuro et al. [17]	72	F	L	Other than LLL	Core biopsy	Supine	19-22
	59	F	R	Other than LLL	Core biopsy	Prone	19-22
Ishii et al. [18]	80	M	Not specified	Not specified	Core biopsy	Supine	18 or less
	71	M	R	Other than LLL	Core biopsy	Prone	19-22
Jang et al. [19]	74	M	R	Other than LLL	Core biopsy	Supine	Not specified
	65	M	L	Other than LLL	Core biopsy	Supine	Not specified
Kawaji et al. [20]	77	M	R	Other than LLL	Core biopsy	Supine	18 or less
Kazimirko et al. [21]	65	M	R	Other than LLL	Needle aspiration	Decubitus	22 or less
Khalid et al. [22]	76	M	L	LLL	Core biopsy	Prone	22 or less
Kuo et al. [23]	74	M	R	Other than LLL	Core biopsy	Supine	18 or less
Matsuura et al. [24]	74	M	Not specified	Not specified	Core biopsy	Not specified	Not specified
Mokart et al. [25]	57	M	L	LLL	Core biopsy	Prone	Not specified
Oh et al. [26]	80	F	R	Other than LLL	Core biopsy	Supine	18 or less
Ornelas et al. [27]	70	F	L	Not specified	Core biopsy	Supine	Not specified
Shroff et al. [28]	50	M	R	Not specified	Needle aspiration	Supine	Not specified
Smit et al. [29]	71	M	L	Not specified	Core biopsy	Prone	18 or less
Sun et al. [30]	53	F	L	LLL	Core biopsy	Prone	18 or less

Statistical Analysis

Information regarding individual patient characteristics, lesion location, complications, and outcomes were analyzed from each case report or literature source used. These data were expressed as a percentage. Variable differences were compared. A p-value of <0.05 was considered statistically significant. Nominal variables were compared using a Fisher’s exact test.

Results

Figure 1 depicts the algorithm used to filter the cases used in the study. A total of 31 cases from 26 literature were ultimately utilized (Table 1). In the data, biopsies of a left lower lobe (LLL) lesion were most significantly associated with cardiac arrest from any cause (p=0.001). Of the patients who suffered an air embolism from a LLL biopsy, 80% also experienced cardiac arrest (p<0.001). Of those patients who experienced cardiac arrest from an CAE after a LLL biopsy, 75% ultimately expired. At the same time, interventions in other lobes were associated with 100% survival. Of the patients who did not experience cardiac arrests after LLL CAE, 4.35% expired. Predictors of patient survival were strongly correlated with patient-specific risk factors (Table 2). Biopsy of the LLL and cardiac arrest were the two biggest factors influencing overall patient survival (p=0.010, p<0.001, respectively). Age, biopsy method, patient position, air location, or employment of hyperbaric oxygen did not statistically influence survival. Although not statistically significant, the culprit artery for the vast majority of CAE was the right coronary artery (RCA). 

**Table 2:** Factors influencing survival in CT-guided PLB. CT: computed tomography; LLL: left lower lobe; PLB: percutaneous lung biopsy; RCA: right coronary artery.

Factor	Level	Expired	Survived	p-value
N		7	24	
Age, mean (SD)		64.86 (10.62)	66.20 (9.88)	0.78
Sex	Male	2 (29%)	5 (21%)	0.64
	Female	5 (71%)	19 (79%)	
Biopsy lesion	Left	5 (71%)	8 (36%)	0.19
	Right	2 (29%)	14 (64%)	
Lobar location	LLL	3 (100%)	2 (12%)	0.01
	Other than LLL	0 (0%)	14 (88%)	
Biopsy method	Core biopsy	5 (71%)	20 (87%)	0.57
	Needle aspiration	2 (29%)	3 (13%)	
Patient position	Decubitus	2 (29%)	2 (10%)	0.36
	Prone	3 (43%)	8 (38%)	
	Supine	2 (29%)	11 (52%)	
Biopsy needle	18 g or less	3 (60%)	9 (47%)	0.32
	19-22 g	1 (20%)	9 (47%)	
	>22 g	1 (20%)	1 (5%)	
Cough	No	3 (50%)	14 (64%)	0.65
	Yes	3 (50%)	8 (36%)	
Air location	RCA	4 (57%)	19 (79%)	0.33
	Other than RCA	3 (43%)	5 (21%)	
Hyperbaric oxygen	No	5 (71%)	17 (71%)	1
	Yes	2 (29%)	7 (29%)	
Cardiac arrest	No	1	22 (99%)	<0.001
	Yes	6	2 (8%)	

When assessing lobar location with cardiac arrest using a Fisher's exact analysis, 80% of patients who experienced cardiac arrest had intervention in the LLL. Only one patient with intervention in the LLL did not have cardiac arrest (Table 3). Of all patients who expired, 85.71% had cardiac arrests. For patients who survived, 8.33% had cardiac arrests.

**Table 3:** Percentage of LLL biopsy patients suffering cardiac arrest from CAE using Fisher's exact analysis. CAE: coronary air embolism; LLL: left lower lobe. Fisher's exact=0.001.

Lobar location	Cardiac arrest present	Cardiac arrest not present	Total
LLL	4 (80%)	1 (20%)	5
Other than LLL	0 (0%)	14 (100%)	14
Total	4	15	

In patients with confirmed CAE, three patients (60%) expired and two patients (40%) survived after intervention in the LLL. At the same time, of the patients who survived CAE, 14 patients (87.5%) had intervention in a lobe other than the LLL, and two patients (12.5%) had intervention in the LLL (Table 4).

**Table 4:** Mortality of patient undergoing PLB by location. LLL: left lower lobe; PLB: percutaneous lung biopsy. Fisher's exact=0.010.

Lobar location	Expired	Survived	Total
LLL	3	2	5
Other than LLL	0	14	14
Total	3	16	19

Discussion

Pathophysiology and Epidemiology

The initial air embolism most likely results from the iatrogenic introduction of gas bubbles into the bloodstream. Three possible causes include communication between pulmonary veins and atmosphere, bronchopulmonary venous fistula (BPVF) or other air-containing spaces and pulmonary veins attributed to needle puncture, and air from pulmonary arterial circulation traversing through the pulmonary microvasculature and reaching pulmonary venous circulation [31]. In the prone position, it has been hypothesized that the entry of air into a BPVF or an alveolar pulmonary venous fistula (APVF) is complimented by the increased hydrostatic pressure of the venous vessels when a biopsied lesion is superior to the left atrium [31]. An increased external atmospheric to central venous pressure (CVP) gradient also promotes the diffusion of air through the pulmonary vasculature and can be exacerbated by hypovolemia and inspiration [32]. Although patients receiving an outpatient lung biopsy are more likely to be euvolemic, it has been hypothesized that up to 40% of the general population have a sub-atmospheric CVP at baseline [33].

Once in central circulation, the air embolism can theoretically pass through the left side of the heart, into the aorta, and retrograde through the coronary arteries during diastole. As a preventative measure, placing a patient in the ipsilateral-dependent position, with the lesion below the left atrium, has been shown to decrease the incidence of systemic air embolism [34]. A recent meta-analysis further found that subsolid lesions in the LLL, lesions superior to the left atria, and the presence of pneumothorax and hemorrhage were associated with an increased risk of systemic air emboli [35]. It also concluded that emphysema, air location, and cough were related to patient symptoms, with the presence of symptoms significantly influencing patient outcomes [35].

This systematic review revealed the RCA as the most common location of air than other coronary arteries or branches; however, this finding was not significantly associated with cardiac arrest or patient expiry (Table 5). Even though the exact pathophysiology is unknown, we postulate that the anterior anatomical location of the RCA origin allows air to pass through the RCA more commonly than the more posteriorly located left coronary artery (LCA). When present, we found that coronary air emboli from a LLL biopsy not only resulted in cardiac arrest in 80% of patients with CAE (Table 3) but was also associated with a 60% overall mortality rate (Table 4).

**Table 5:** Correlation of patient-specific variables with cardiac arrest after CAE from CT-guided percutaneous lung biopsy. CAE: coronary air embolism; CT: computed tomography.

Factor	Level	Cardiac arrest (not present)	Cardiac arrest (present)	p-value
N		23	8	
Age, mean (SD)		67.087 (10.0856)	62.5 (9.02378)	0.27
Sex	Male	4 (17%)	3 (38%)	0.33
	Female	19 (83%)	5 (62%)	
Biopsy lesion	Left	8 (38%)	5 (62%)	0.41
	Right	13 (62%)	3 (38%)	
Lobar location	LLL	1 (7%)	8 (100%)	0.001
	Other than LLL	14 (93%)	0 (0%)	
Biopsy method	Core biopsy	19 (86%)	6 (75%)	0.59
	Needle aspiration	3 (4%)	2 (25%)	
Patient position	Decubitus	2 (10%)	2 (25%)	0.73
	Prone	8 (40%)	3 (38%)	
	Supine	10 (50%)	3 (38%)	
Biopsy needle	18 g or less	9 (50%)	3 (50%)	0.64
	19–22 g	8 (44%)	2 (33%)	
	>22 g	1 (6%)	1 (17%)	
Cough	No	14 (67%)	3 (43%)	0.38
	Yes	7 (33%)	4 (57%)	
Air location	RCA	18 (78%)	5 (62%)	0.39
	Other than RCA	5 (22%)	3 (38%)	
Hyperbaric oxygen	No	16 (70%)	6 (75%)	1
	Yes	7 (30%)	2 (25%)	
Outcome	Expired	1 (4%)	6 (75%)	<0.001
	Survived	22 (96%)	2 (25%)	

Clinical Manifestations

Clinical manifestations of CAE (and SAE) are the result of ischemia and end-organ damage within the affected circulation, and the diagnosis of CAE relies heavily on the clinical suspicions of the healthcare provider. With symptoms often being non-specific and ranging from seizures and altered mental status to dyspnea, tussis, and chest pain, the diagnosis of CAE or SAE can be easily overlooked [32]. Clinical signs that may allude to a potential air embolism include cyanosis, tachypnea, tachycardia, and wheezing [36]. When affecting coronary circulation, air emboli may cause chest pain, hypotension, bradycardia, arrhythmias, ST-elevation myocardial infarction (STEMI), with or without atrioventricular heart block, on the surface electrocardiogram (ECG), and hemodynamic compromise may manifest within seconds of symptom onset [37,7]. Atrial flattening, ventricular dilation, and extrasystoles are harbingers of cardiovascular collapse and are physical findings that may potentially be detected by point-of-care ultrasound (POCUS) with concurrent telemetry monitoring [38]. Although potentially severe and debilitating, symptoms of CAE can resolve as quickly as they present with overall resolution within 5-10 minutes [39].

Imaging

Several imaging modalities are available to aid in the diagnosis of CAE. A plain film chest radiograph can usually be obtained within an appropriate amount of time and can rule out other causes of chest pain, shortness of breath, and shock, for example, tension pneumothorax, hemorrhage, consolidation, severe aortopathy, and pulmonary edema. Although CAE cannot be definitively diagnosed on a plain chest X-ray (CXR), there have been cases depicting increased right ventricular and pulmonary arterial lucency in the setting of venous air embolism (VAE) [40]. Significant air burden with intracardiac valvulopathies (mitral/aortic valve stenosis or insufficiency) or low-flow states may theoretically allow for an air embolism to be appreciated in the left atrium (LA), left ventricle (LV), or aorta (AO) as an increased lucency.

Although sensitivities and specificities could not be obtained, the literature generally supports the use of CT and MRI for the diagnosis of CAE. It should be noted, however, that air emboli may be diagnosed clinically if suspicion is high [32]. On CT imaging, an extremely hypoattenuated lesion with Hounsfield Units (HU) reaching -1000 can be identified as air [41]. A lesion with this level of HU within the coronary circulation would be consistent with CAE. Because of very low magnetic susceptibility, air emboli can theoretically be identified by MRI as areas of signal loss, especially on T2-weighted gradient-echo (GRE) images [42]. Although not likely to be present during a CT-guided PLB, both transthoracic echocardiography (TTE) and transesophageal echocardiography (TEE) have been useful in determining the presence of air within the cardiac chambers [43,44].

Treatment

Given the disastrous effects of CAE, initial management should be geared towards education, training, safe practices, and embolism prevention [45]. Other methods that may aid in prevention include patient positioning and ensuring pre-procedure euvolemia. When present, treatment of CAE is largely dependent on symptoms and patient hemodynamic instability. When asymptomatic with small amounts of air, intervention may not be needed [46]. In the event, a patient is symptomatic or hemodynamically unstable, supportive therapies should be initiated until definitive therapy is available. The management of CAE closely mirrors that of significant decompression sickness. Supplemental 100% oxygen is a safe and generally recommended practice, regardless of oxygen saturation [39,47-49]. The effects on tissue distal to the CAE occlusion are notably similar to those of plaque rupture, with areas of hypoperfusion and hypokinesis readily observable [38]. Regarding the latter, meta-analyses have cautioned against routine supplemental oxygen administration, as oxygen-free radicals contribute to reperfusion injury and no-reflow phenomenon [50-52]. If possible, aspiration of the air should be attempted with the administration of inotropes, positive chronotropic agents, and vasopressors (systemic and/or intracoronary) as needed [47,48]. Forceful saline injection is another method used to decrease air burden, and in severely unstable patients, intra-aortic balloon pumps (IABPs), cardiopulmonary resuscitation (CPR), and direct current cardioversion (DCCV) may be necessary [49]. An additional invasive therapy that has reported success is manual disruption and dislodgement of the CAE by the guidewire during angiography [53]. Penultimately, the physical properties of gas can be manipulated in order to dissolve the embolus. With the size of an air bubble being inversely proportional to atmospheric pressure, hyperbaric oxygen is a safe and frequently used treatment in CAE as well as decompression sickness [54]. Systemic anticoagulation with heparin and lidocaine administration appear to be promising neuroprotective interventions in animal models; however, additional investigations are necessary to determine the neurological and cardiological benefits in humans [55-57].

Limitations

The authors acknowledge several limitations within this study. First of all, the study was limited to one database. Because of the limited number of databases used, there was a limited sample of cases to analyze. Some cases had to be dismissed, as there was not enough information in the parent article. Small sample sizes only allowed two risk factors for patient mortality to be statistically significant. Second, some cases did not confirm CAE with imaging and, therefore, had to be excluded. Third, some air emboli may have been unrecognized due to a lack of patient reporting and/or a lack of patient symptoms. Fourth, given that the foundation of the study was based on case reports, there is a lot of room for interpretation, and some cases may not have been of sound quality. Fifth, this study excluded any type of extra-coronary air embolism, which limited the number of cases that met inclusion criteria. This also likely led to the under-analysis of total cardiac arrests. Thus, mortality rates may be altered from the general population receiving CT-guided PLB. Lastly, this study only evaluated CAE from CT-guided lung biopsies. Other modalities may have variable CAE and mortality rates affecting different pulmonary lobes.

## Conclusions

This review adds to the current evidence significantly associating systemic air with CT-guided PLB of the LLL. As expected, an increased risk of SAE from LLL CT-guided PLB will undoubtedly increase the risk of developing CAE. Similarly, CAE and cardiac arrest from CAE were significantly associated with LLL biopsies. LLL intervention and the presence of cardiac arrest significantly influenced overall mortality post-biopsy. Emphasizing SAE prevention, needle gauge, patient position, biopsy method, air location, and hyperbaric oxygen administration significantly influenced survival after CAE. Should CAE occur, treatment is largely supportive; however, a multi-disciplinary team is often required. In certain cases, intravascular intervention may be necessary or curative. Anatomically, the RCA appears to be correlated with a higher incidence of CAE, but more studies will be needed to further evaluate this finding.
